# The emergence of emotionally modern humans: implications for language and learning

**DOI:** 10.1098/rstb.2019.0499

**Published:** 2020-06-01

**Authors:** Sarah Blaffer Hrdy, Judith M. Burkart

**Affiliations:** 1 Citrona Farms, 21440 County Road 87, Winters, CA 95694, USA; 2 Department of Anthropology, University of Zurich, Winterthurerstrasse 190, 8051 Zurich, Switzerland

**Keywords:** cooperative breeding, ingratiating impulses, language, concern for reputation, inter-subjectivity, conscience

## Abstract

According to the Cooperative Breeding Hypothesis, apes with the life-history attributes of those in the line leading to the genus *Homo* could not have evolved unless male and female allomothers had begun to help mothers care for and provision offspring. As proposed elsewhere, the unusual way hominins reared their young generated novel phenotypes subsequently subjected to Darwinian social selection favouring those young apes best at monitoring the intentions, mental states and preferences of others and most motivated to attract and appeal to caretakers. Not only were youngsters acquiring information in social contexts different from those of other apes, but they would also have been emotionally and neurophysiologically different from them in ways that are relevant to how humans learn. Contingently delivered rewards to dependents who attracted and ingratiated themselves with allomothers shaped their behaviours and vocalizations and transformed the way developing youngsters learned from others and internalized their preferences.

This article is part of the theme issue ‘Life history and learning: how childhood, caregiving and old age shape cognition and culture in humans and other animals’.

## 1. Setting the Plio-Pleistocene stage

Apes with the life-history attributes of *Homo sapiens* could not have evolved unless male and female allomothers had helped mothers care for and provision offspring. We refer to this as the ‘Cooperative Breeding Hypothesis’ [1,2]. Across the animal world, breeding systems characterized by group members other than parents (alloparents) helping parents to care for and provision offspring have evolved many times in social insects, in 9% of 10 000 species of birds [3], and in perhaps 3% of 4500 species of mammals. The prevalence of allomaternal care tends to be higher among social carnivores [4], and higher still among primates, where females or males other than the mother care for and *at least minimally provision* offspring in more than 30% of species [5,6]. Extensive alloparental in addition to parental provisioning, however, is only found among humans and in a distantly related subfamily of New World monkeys, the Callitrichidae, containing marmosets and tamarins.

Various circumstances conducive to the evolution of cooperative breeding pertained among hominins in Plio-Pleistocene Africa [7]. These included: cohesive social groups containing close relatives; production of increasingly costly, slower maturing young; increasing reliance on hunting and extractive foraging such that immatures began to depend on adults to acquire or process food for them and to facilitate their learning appropriate skills [1,4,8–12]; and importantly, ecological instability [13,14].

Unpredictable rainfall against a background of increasing aridity almost certainly figured in the emergence of shared provisioning among early hominins [12,15–18]. In spite of recurring periods of food shortage accompanied by high child mortality, hominin mothers in the line of bipedal apes leading to *H. sapiens* were producing slower maturing, increasingly large-brained, energetically more costly offspring, yet also beginning to produce infants after shorter intervals. How? Along with others, we hypothesize that by 2 Ma with *Homo erectus*, hominin mothers increasingly relied on assistance from other group members to supplement offspring who even after weaning remained years from nutritional independence [9,19–23]. Earlier weaning meant mothers could resume cycling, conceive again and reproduce faster. This is a conservative interpretation. Others propose the emergence of allomaternal assistance as early as *Australopithecus* [24] or *Ardipithecus* [25].

Over time, stacking of closely spaced dependent offspring would further intensify reliance on allomaternal provisioning. By the Pleistocene, we suspect that hominins were adopting even more flexible residence patterns than those found in the other great apes [26], with ‘multi-local’ residence patterns beginning to resemble those typical of twentieth century hunter–gatherers [27,28]. Greater female autonomy of movement and the emergence of pair-bonds (why they emerged being a topic still debated) would increase chances that probable fathers and matrilineal kin resided nearby [29].

Greater postpartum tolerance on the part of ordinarily possessive ape mothers coevolved with growing, albeit still facultative, neurophysiological responsiveness on the part of fathers and other allomothers increasingly motivated to care for immatures [25,30,31]. The more dependent upon allomaternal assistance primate mothers are, the more sensitive they become to cues of social support, especially postpartum [21,32]. Compared with the reflexive protectiveness and possessiveness typical of all but the most stressed or inexperienced great ape mothers, who carry comatose or even dead infants for days, postpartum commitment of these hominins would likely have been more conditional. Across traditional societies, mothers are known to abandon at birth infants considered defective as well as adjust parental investment in line with social and ecological circumstances [21, ch. 12 and 13], [33,34].

**Figure 1.**
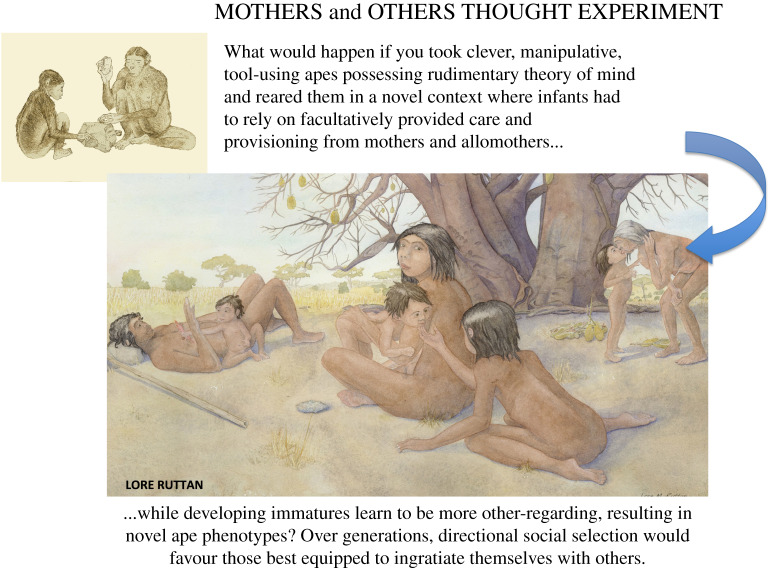
This thought experiment traces dual ontogenetic and evolutionary processes set in motion when mothers in the line leading to the genus *Homo* began to rely on alloparental care and provisioning to rear increasingly costly, sometimes more closely spaced, offspring. Intermittent behavioural conditioning would encourage youngsters to repeat and refine facial expressions, attitudes and vocalizations most likely to appeal to potential caretakers. This would lead to the expression of otherwise latent potentials and the formation of novel neural networks. Over generations, these quite novel ape phenotypes would be exposed to directional social selection favouring youngsters best at ingratiating themselves with others, setting in motion a causal chain of adaptive evolution that began with development [35]. Art by L. M. Ruttan and used with permission. (Online version in colour.)

Infants who could no longer count on being the sole priority of a single-mindedly dedicated mother had to elicit and maintain maternal commitment while also attracting, and increasingly ingratiating themselves with, others. Rewarded when they succeeded, over the course of development infants learned to express otherwise latent sociocognitive potentials. As we use the term, ‘ingratiating behaviours' refer to anything an infant does to increase his or her chances of being chosen as the object of beneficent attention (including provisioning), where offspring best at ingratiating themselves with others would be most likely to survive. Over the course of development, these youngsters learned to monitor and be interested in the intentions, thoughts and feelings of others, and even internalize their preferences. Over generations, youngsters best at doing so would be more likely to survive, resulting in populations of apes emotionally very different from their ancestors (figure 1).

In this contribution, we reconstruct how growing up in such a social environment may have impacted dependent immatures along with the cascading consequences this would have had on uniquely human forms of learning and language. We start by asking: How would dependent immatures respond to the challenges of eliciting vital, but facultatively proffered, maternal and allomaternal attentions? What would this little ape need to do? Since none of us can go back in time to observe how early hominin infants would have behaved, we draw on evidence for how non-human ape and modern human babies responded in ‘virtual ‘experiments with and without allomaternal care to test assumptions made here about how hominin infants would have behaved with it [2], as well as on evidence from callitrichid monkey infants reared in a cooperative context.

Although inevitably speculative, this reconstruction is informed by both comparative evidence and a growing understanding of the survival challenges hominin infants confronted. As our point of departure though, we begin with callitrichid monkeys, the only extant primates besides humans with extensive alloparental in addition to parental care and provisioning of infants. Callitrichids face similar challenges to those hominin infants would have although they do so endowed with far smaller brains. Callitrichid mothers customarily give birth to twins, and owing to postpartum oestrus are immediately pregnant again [36,37]. They are thus lactating and gestating at the same time, and the new set of offspring is born before the previous one is fully independent. This costly reproduction is only possible because other group members act as allomothers and help by carrying infants, protecting them and substantially provisioning them [38]. Like human mothers, callitrichid mothers are highly tolerant towards other group members interested in carrying their babies, and they adjust their maternal investment relative to how much help is available [21,39]. Helpers provisioning immatures exhibit highly prosocial motivations [38,40]. Within the social group, all members are characterized by high levels of tolerance and prosociality compared with independently breeding primates [41]. Youngsters born to hominin mothers who, when conditions permitted, similarly produced more closely spaced young (in ‘as-if’ litters), confronted challenges similar to those callitrichid twins face. Thus did infants in distantly related species converge upon similar modes of eliciting succour from allomothers as well as mothers. But how might such solutions be extended and transformed if dependent immatures were endowed with much more powerful ape cognitive systems to begin with?

## 2. The hominin infant's to-do list

### (a) First order of business: appeal to mother

Although born with open eyes, able to blurrily seek the eyes of someone else, newborn apes are otherwise altricial, utterly dependent on others for warmth, protection, locomotion and food [42,43]. Fortunately, apes enjoy the built-in mammalian advantage of being born to a mother who during gestation was suffused by hormones lowering her threshold for responding to sounds and smells of a warm, wriggling, fluid-covered baby. If only the newborn can make it to maternal nipples, root, suck and stimulate lactation, ensuing surges in oxytocin and prolactin further enhance her nurturing impulses. With lactation underway, even an inexperienced first-time ape mother becomes increasingly bonded to this newcomer. But over the course of hominin evolution, increasing maternal reliance on allomaternal assistance would have rendered mothers increasingly sensitive to cues of available social support. In the absence of social support, the commitment of even the most experienced human mother falters (see especially [21,32,44]).

Over evolutionary time, more contingent commitment preadapted human mothers to become more discriminating than other apes. In addition to their parity, prior experience, physical condition and likely social support, mothers began to respond to specific physical attributes of each infant. Consciously or not, each costly infant was in competition not only with still nutritionally dependent older siblings, but also with subsequent infants a mother might bear under ecologically more opportune conditions if only she retrenched upon investment in this one, or bailed out altogether, and resumed cycling sooner. Over the course of the Pleistocene (perhaps earlier?), hominin babies came under increasing pressure to look good and sound vigorous right at birth, advertising that they were full-term, robust, good bets for survival, worth further investment. This challenge may help explain why, over the course of hominin evolution, fetuses began to stockpile adipose tissue at an unlikely time, just prior to squeezing through what were becoming increasingly narrow birth canals. By now, human neonates are born much fatter than other apes (*ca* 10–14% body fat contrasted with 3–4% for chimpanzees). No doubt, an extra dollop of energy was initially beneficial for thermoregulation and fuelling fast-growing brains, contributing to the emergence in mothers of sensory biases favouring plump babies [45]. Over time, plumpness may also have come to serve as an infantile equivalent of sex appeal, seducing mothers into embarking on a long, exorbitantly costly, endeavour [21, ch. 21].

Once increasingly discriminating hominin mothers began to notice associations between birth weight and later outcomes, one of evolution's more consequential self-reinforcing feedback loops would be underway. With runaway social selection (*sensu* West-Eberhard) for neonatal plumpness underway, extra energy stores became available for brain development, in turn rendering allomaternal provisioning even more essential. Over generations, allomothers as well became attuned to cues of neonatal viability, adjusting levels of commitment accordingly [32].

This brings us to the hominin infant's *second order of business*.

### (b) Attract others

Because hominin mothers as well benefitted from allomaternal assistance, it behoved females to reside near trusted others, matrilineal kin and/or adult males who were probable fathers. Unlike exceedingly possessive great ape mothers, mothers in traditional human societies not only tolerate, but often encourage postpartum contact with infants. Whether this shift was due to innately more tolerant temperaments, to greater autonomy in selecting whom to live among, or both, is not clear. In any event, maternal tolerance facilitated intimate exposure of trusted group members to infantile smells and sounds, generating maternal-like affiliation-promoting neurophysiological transformations in male and female allomothers alike [1,46–48]. Among humans, only a few hours of intimate contact with grandparents is sufficient to produce surges in oxytocin and rearrange priorities [49]. (Among marmosets, similarly, oxytocin increases not only in mothers, but in all group members after the birth of new babies [50].)

But other factors also enter in, including the allomother's own physical condition and past caretaking experiences, and alternatives available, as well as the infant's vulnerability and level of need. Facing this uncertainty, cues from infants themselves to elicit allomaternal involvement would again be paramount. Over time, human allomothers become sensitive to the same viability cues that mothers respond to. Magnetic resonance images of the brains of modern humans reveal that even nulliparous women find the faces of plump, healthy-looking (read full-term) babies ‘cuter’ and more rewarding to look at [51]. Not only physical viability cues, but also behavioural interactions, will attract carers. Humans are born with neurological capacities comparable with those of other apes, but differences soon emerge [52]. At birth, both chimpanzee and human newborns seek out eyes, are capable of mutual gazing, and caught just right, may imitate someone else's outstretched tongue or other facial expressions [53]. Both species exhibit reflexive ‘fairy smiles’, soon to be replaced by more open-eyed ‘social smiles’ in response to someone else. Among newborn chimpanzees however, that someone else would always be their own mother [54]. In traditional human societies, however, blearily gazing newborns engage a wider audience [22,33]. Over time as infants grow accustomed to and learn to trust specific allomothers, the sort of emotional bonds primate infants forge with mothers prove sufficiently elastic to encompass multiple attachment figures, an average of six attachment figures among the Central African Aka hunter–gatherers Courtney Meehan studied [55]. It is not known whether human infants are more prone to forge multiple attachments than are other apes if cared for by both mothers and others because the latter virtually never are.

A challenge unique to immatures of cooperatively breeding primates is that they are not in continuous contact with their carer. This can be buffered by proactive caring motivations by adults, as in callitrichids, where group members check babies on carriers, eager to take over when necessary [56], or proactively announce that they have found food and are ready to share it. Such provisioning is different from food sharing patterns in other primates, where at most, immatures are passively tolerated when taking their mother's food [57,58]. Nevertheless, without being in constant body contact with a single carer, cooperatively breeding immatures face the chronic risk of being overlooked.

Among apes such as chimpanzees, gorillas or orangutans, newborns constantly held by mothers in direct skin-to-skin contact have less need to smile or vocalize. Calling would only be useful if separated, or later, at weaning, as youngsters object to maternal rejections. Otherwise, low, scarcely audible sounds make more sense than loudly advertising to predators ‘vulnerable baby here!’ Among primates with shared care, life tends to be noisier. Infants need to stay ‘in touch without touch’ and may complain to prompt maternal retrievals. Infant langur monkeys spend up to 50% of their first day of life being held and carried by females-other-than-mothers, calling incessantly [59].

Life is even noisier in animals with biparental and alloparental provisioning, where babies beg for treats. This correlation is best documented in birds [60], but it also holds for callitrichids and humans, who fall among the most voluble of primates [61]. Vocalizing starts early in marmosets and tamarins, becoming more frequent and specialized over the course of development. Begging calls spike around weaning, when allomaternal provisioning is most critical [57,62]. But these infants not only are noisy beggars, but also engage in babbling-like behaviors, by producing repetitive, random-sounding streams of elements of adult vocalizations that can last for more than a minute. This babbling-like behaviour comes with likely cost because it is noisy and makes infants conspicuous to predators. It peaks around weaning, when allomaternal provisioning is most critical, and turns out to be an effective attention getter, as adults are more likely to approach and interact with immatures that are babbling [63].

Something similar goes on in humans, with the onset of babbling around the time (in hunter–gatherers at least) when allomothers begin providing edible treats (discussed below). Within weeks of birth, human infants emit engaging noises. Learning progresses more rapidly if infants notice others reacting. By 10 weeks some actually take turns vocalizing. The sound of a baby laughing generates an especially powerful stimulus, audible at some distance and signalling emotional engagement [64,65]. As babies put two and two together, conditioning plus early glimmerings of inter-subjectivity [66] lead them to incorporate sensory biases and preferences of potential caretakers into their own expanding repertoires for ingratiating themselves with others [67]. By nine months, little humans go out of their way to be helpful. Human youngsters clearly care about what others think of their performance [68].

### (c) Vocal control and more flexible vocalizing

Old World monkeys and apes are sophisticated communicators. Vervet monkeys, for example, emit one kind of call to alert group-mates of raptors, a different alarm call for terrestrial enemies. They are also sensitive to context, taking into account who is listening and who is out of range, modifying calls accordingly [69]. Apes, particularly chimpanzees and bonobos, also make extensive use of hands and arms to communicate what they want, extending an arm palm up when requesting something. Even so, their vocal repertoires never achieve the richness, sophistication and flexibility of their gesturing [70]. Rather, non-human apes seem surprisingly limited in the kinds of vocalizations they emit [71], a marked contrast with humans, who early in development increase vocal control and start to build larger and more flexible vocal repertoires [72]. So how did this get started?

One important element developed elsewhere concerns the challenges that adults, rather than the immatures of cooperative breeders, are confronted with [61,73]. They face increasing necessity to coordinate their activities, such as for instance infant transfers, with others and their prosociality motivates them to share not only food with others, but also information that is useful for them. Vocal communication is a prime candidate to provide a solution to exactly these type of challenges, and accordingly, the large vocal repertoires of cooperatively breeding birds are driven by an increase in contact and alarm calls [60]. But a critical element is added by immatures, who grow up in an increasingly voluble environment.

From a comparative perspective, it seems clear that shared care with babies carried by others increases vocalization frequency. Allomaternal provisioning and contingent reinforcement raises the stakes, with begging leading to more calling, especially if immatures have to compete for rewarding attentions [60,74]. Through the expression of otherwise latent capacities and their subsequent shaping, attention-getting and begging set the stage for selection to favour enhanced vocal control accompanied by goal-oriented shaping of acoustic structure. Indeed, as in marmosets, vocal development in humans occurs earlier than motor development [75]. By contrast, infants in constant close contact with single-mindedly dedicated mothers (as in independently breeding species such as chimpanzees) would more often be called upon to cling than cry.

Experiments with marmosets undertaken by Asif Ghazanfar and his team demonstrate how contingent responsiveness by caretakers generates turn-taking and also speeds development of specialized, more mature-sounding calls [76]. For 40 min a day during the first two months of life, each of a pair of marmoset twins was separated from their parents and allowed to call. Whereas one twin was provided consistent feedback from taped parental calls, the other twin received less consistent feedback. The more reliable the feedback, the more rapidly infants progressed from the coarse, random-sounding vocalizations typical of immatures to cleaner, more tonal, adult-like *phee* calls [76,77]. By two to three months of age, their utterances resembled the turn-taking ‘conversations’ human babies engage in with their caretakers. Chow *et al*. [78] further showed that parents actively intervene when immatures make typical mistakes while learning to engage in turn-taking. If immatures get timing wrong and ‘interrupt’ their parents, parents add an extra break before responding. When immatures respond with a wrong call type, the parents themselves interrupt them with the correct *phee* call. In another example of convergent evolution between human cooperative breeders and these tiny-brained, distantly related New World monkeys, Takahashi and colleagues noted that infant marmosets responding to contingent reinforcement rely on one of the same circuits to guide their *phee* calls that humans use in speech. The patterning of FoxP2 expression in marmosets' cortico-striatal circuit turns out to be analogous to that in both humans and songbirds [77]. Moreover, a role of oxytocin has recently been proposed for the social motivation and evolution of vocal learning and language [79], which is consistent with the increase of oxytocin in all group members after the birth of marmoset immatures [50] and its link with prosociality among group members [80].

Observations of golden lion tamarins (*Leontopithecus rosalia*) in Brazil illustrate how these increasingly complex, two-way conversations function in natural habitats [81]. Solitary adults traverse the treetops hunting for spiders, insects, and small frogs, prying prey from inside holes or tangled foliage. Youngsters learn to locate, stalk, and dexterously extract and dispatch struggling, sometimes biting or stinging, prey. Learning takes time and practice as immatures grow more adept at responding to adults volunteering prey. Deliveries peak near the end of weaning, when up to 90% of their diets are provided by (mostly male) allomothers [81]. Food transfers are often initiated by youngsters begging. But when adults locate food, provisioners too emit staccato ‘food-offering calls’. Mothers, probable fathers, and other helpers extract the food and call the infant to come and get the food out of their hands. As prey-catching efficiency improves, but before youngsters reach adult proficiency, mentors switch from ‘come and get it’ calls, to ‘hey, look here’. Adult calls direct older immatures' attention to a particular patch of substrate where prey have been detected. The finder then waits nearby while the young locate and extract it for themselves. Such adjustment of adults to immature skill levels has also been found in other callitrichids in captive studies (cotton-top tamarins [82], common marmosets [78,83]). As infants increasingly associate an allomother's particular call with a particular reward, they register regularities in how others respond to particular sounds they themselves make.

Opportunities to link own vocal productions to regularities in how others respond to them are particularly evident for babbling (humans) or babbling-like behaviours (callitrichids). In humans, babbling emerges spontaneously at around five to seven months, about the same time as the emergence of milk teeth, which among hunter–gatherers often coincides with allomothers beginning to offer premasticated and other (sometimes ‘kiss-fed’) treats to infants [1]. At some level (consciously or not), children recognize that babbling attracts rewarding attention. This may help explain why older children revert to ‘baby talk’ after the birth of a younger sibling (S. B. Hrdy 2019, personal observation). Interestingly, babbling in marmosets not only attracts carers, but also speeds up the acquisition of adult-like forms of vocalizations. Moreover, babbling similarly can resume and spike among juvenile marmosets following the birth of new infants in their group [84]. Apparently, the same message is being conveyed: ‘pay attention to *me*!’

‘If babbling changes adult behaviour in predictable, infant-oriented ways’, as Goldstein and colleagues propose, ‘infants should be able to recognize changes in others' actions as a result of their vocalizations’ [74, p. 8034], launching more goal-oriented vocal control. As with other apes, humans are born with limited motor control over articulation, but beginning around six months humans gain increased vocal control, including more tongue involvement with vocal tracts continuing to develop over the first 15 months, contributing to greater vocal flexibility and larger vocal repertoires in humans than other apes [72]. Impressed by the coincidence in timing between increased vocal control and the transition from baby-calls to babbling-like streams of consonants and vowels, Klaus Zuberbühler makes a compelling case that increased control derived from hominin infants' need to attract allomaternal attentions [72, pp. 71, 77–79]. Although ‘babbling’ is widely assumed to have first emerged in human children in preparation for the acquisition of spoken language, akin to Mother Nature adding training wheels on a bicycle, it makes more sense (and is far less teleological) to consider the initial emergence of traits like babbling as byproducts of infantile needs to attract carers. A key innovation here was increased motor control over articulation. Once vocalizations become subject to voluntary control they can be shaped via conditional reinforcement, critical preadaptation for the eventual evolution of spoken language.

### (d) Incorporate others' preferences

These novel capacities emerged within broader sociocognitive contexts where apes were already endowed with rudimentary Theory of Mind [85–87], already using rich gestural repertoires [70], already employing tools and devising new modes of extracting food. By the Late Pleistocene, selection pressures from a range of new subsistence and socioecological challenges also favoured greater inter-individual coordination [38,88–90]. It is within this broader context that recent proposals need to be understood that link cooperative breeding to the evolution of enhanced capacities not only for joint attention [91] but also for language [61,72,92].

Social selection favouring more flexible communication coincided with other coevolving feedback loops. But, by themselves, neither larger brains nor increased uses for cooperation are sufficient to explain the evolution of language. As psychiatrist Peter Hobson reminds us ‘before language there (had to be) something else … that could evolve in tiny steps … that something else was *social engagement with each other*. The links that can join one person's mind with the mind of someone else are, especially to begin with, emotional links' ([93, p. 2]; emphasis in original). But what about the foundational steps? Klaus Zuberbühler's speculations point us in a promising direction: ‘Once vocal control has evolved to help infants secure care, it is only a small step to producing utterances in context-specific ways’ [72, p. 80]. But, Zuberbühler adds, such a transformation ‘may only be possible against a background of other psychological skills, such as the ability to share intentions and attention [94], and well-developed comprehension’ [72, p. 80]. He expands on Tomasello's insight regarding a ‘major difference’ [95] between humans and other primates, involving (as Tomasello would later phrase it) capacities for ‘intersubjective sharing’ [96, pp. 121–122].

Eagerness to ingratiate themselves with others would be enhanced by allomaternal care, an interpretation consistent with observations of captive chimpanzees who, when co-reared by responsive human caretakers (allomothers of a different species) as well as their mothers, become more eager than wild chimpanzees are to engage in targeted helping of others ([42,97]; reviewed in [2]). Even though human-tended chimpanzees do not acquire spoken language and other distinctively human traits, they nevertheless develop greater concern for the intentions and goals of others, learning the power that particular gestures, facial expressions and utterances exert on others. For example, human-reared chimpanzees point to what they want in ways that wild apes almost never do [87]. The expression of such interactive potentials in ape phenotypes would increase opportunities to share and increase effectiveness of helping. Over the course of human evolution, such opportunities may have increased selection favouring neuroendocrine systems conducive to prosocial responses, including the increasingly ‘dopamine dominated’ striatal systems being documented by palaeontologists working in concert with neuroscientists [25]. Interactions with processes that opened parental neural care systems to a wider range of social stimuli might have resulted in more unsolicited food sharing and general prosociality [31]. Apes who needed to be more interested in the preferences of others would also find it more emotionally rewarding to do so. This chain of admittedly speculative reasoning brings us to a key component to the hominin infant's to-do list.

### (e) Add psychological dimensions to *Kindchenschema*

Like all other catarrhine primates, apes in the line leading to the genus *Homo* would grow up keenly aware of kin ties, alliances, statuses and friendships as well as competencies and reliability of group-mates [6,98]. The quantitative skills and manipulative capabilities of a chimpanzee or orangutan fall in the same ballpark as those of two-and-a-half-year-old humans. They too exhibit rudimentary capacities for theorizing about what others know [85,86,99]. Our Last Common Ancestors with these apes were already beginning to register what others intended or wanted.

So imagine such an ape growing up reliant on the competencies and motivations of others. If contingent reinforcement from allomothers encourages turn-taking and speeds up acquisition of adult vocalizations in tiny-brained marmosets with only minimal awareness of what another marmoset knows [61,100], how much more sensitive to the thoughts and preferences of others would apes already attuned to the thoughts and intentions of others become? Contingent allomaternal responses generate new psychological dimensions to *Kindchenschema* as apes growing up this way are conditioned to become more aware that others have preferences worth appealing to.

Youngsters would be conditioned not only to monitor the intentions of others, but increasingly to probe their thoughts and feelings so as to better conform to their preferences. Over time, learning which facial expressions, sounds or conversational rhythms result in solicitude would mature into more sophisticated understanding of how others perceived their own intentions, behaviours, and thoughts and to begin to care about their ‘reputations’.

## 3. New dimensions to social learning

### (a) Expanded avenues

All apes are endowed with inordinate behavioural flexibility along with aptitudes for manipulating objects and imitating others. In the case of chimpanzees and orangutans, knowledge about what to eat, where to find and how to process it, is transmitted vertically during 5–8 years of intimate association with one other trusted individual, their mother. Mothers set the stage for socially induced independent practice [58,101,102] or, as in the case of chimpanzee nut-cracking, very occasionally make helpful adjustments [54,103]. The processes by which little apes copy and learn from others are however primarily self-initiated [104]. Provisioning and shared care broadens this initial context for social learning.

From an early age, youngsters with shared care observe a wider range of role models. For example, cooperatively breeding magpie jays with many helpers become more adept at harvesting arthropods than jays growing up with few [105]. Furthermore, demonstrators among cooperative breeders tend to be more prosocial, even deliberately helpful [19,106]. Recall that golden lion tamarin allomothers (often probable or possible progenitors) provide the majority of food for nearly weaned infants. First, adults call when they have food to offer, but with older immatures, they call them to places where prey is hidden and the immatures have to do the extraction themselves. As performance levels plateau, food calls cease. Rapaport [81, p. 746] compares the progressive, developmentally sensitive behaviours and vocalizations tracking the needs and skills of youngsters that allomothers use to provide foraging assistance to ‘teaching-like’ behaviour (cf. [107] for cooperatively breeding meerkats).

Incorporating situation-dependent vocalizations enhances effectiveness of demonstrations while contingently delivered food rewards rivets the attention of mentees. Anyone who has ever tried to habituate wild creatures, or even skittish domestic ones, know that food rewards provide the quickest short-cut to taming or training them. Now add to this prosocial–provisioning–vocalizing mix mentalizing mentees eager to accommodate their mentors. Possibilities for information transfer expand exponentially.

### (b) Emotionally modern and mentalizing mentees

Even in the absence of detectable Theory of Mind, tiny-brained marmosets prove remarkably prosocial, sharing food or rushing to assist others. Marmosets coordinate with others to crack open tough fruits. They take babies from mothers then voluntarily return them for nursing [19,37,41]. Tamarins even use guided demonstrations accentuated by vocalizations to transmit age-appropriate information [81]. In this respect, tiny-brained callitrichids converge on something close to what ethnographers studying hunter–gatherers term ‘natural pedagogy’ [108]. Yet even as tamarins adjust demonstrations to the skill level of pupils, they do so without mentalizing what another knows. Marmosets who readily follow the gaze of another individual do so without registering *what* that individual is seeing [100].

So what would happen if instead of the reflexive responsiveness of marmosets, the primates undertaking shared care and provisioning of young were already larger-bodied, bipedal, tool-users possessed of rudimentary Theory of Mind, with brains in the process of doubling from the 400 cm_3_ of chimpanzees or australopiths to the more expansive brains typical of *H. erectus*? And what if novel contexts for social development coincided with new foraging tactics, more valuable food packets, and with these, a raised ‘grey ceiling’ so that over time, whenever—and for whatever reasons—they became favoured, even costlier brains could evolve [5,109]?, Little hominins growing up as cooperative breeders would have opportunities to observe group members of different ages and sexes [9,110,111], trust them as their mothers do, gauge their competencies and intentions, decide who was likely to be helpful or not—something modern humans begin to do from as early as three months old [112,113]. And what if at the same time these infants were beginning to monitor the intentions of others, seeking to conform to their preferences, even beginning to internalize their preferences, and at the same time also developing larger and more flexible vocal repertoires?

Many factors were involved in the evolution of language. Some clearly had to do with the unusual way apes in the line leading to *H. sapiens* were reared. Learning language is a highly social endeavour. Anyone who has ever spent time with babies knows that their mother is not the only person who speaks to them in high-pitched ‘motherese’. It is from eagerly listening to others that youngsters acquire new phonemes and words. Immatures learn new sounds better in the presence of someone else than if by themselves. As young as nine months, babies watching instructive videos more readily discriminate sounds and learn foreign phonemes when another child is present [114].

Not only current interactions but also past experiences with others influence children's readiness to mentalize what someone else knows. When experimenters set up a computer game where 5-year-olds must explain to someone else where to collect a digital prize, they were told that the unseen other (really the experimenters' confederate) was either a toddler or another 5-year-old. Subjects adjusted instructions accordingly. When told their partner was a toddler, subjects spent longer explaining the game than when they assumed the other child was older. The more days between birth and age four that the subject had spent with others in daycare, the readier that child was to take the others' level of understanding into account, mentalizing what they were likely to already know [115].

### (c) Concern for reputation and learning

Within the first year of life, hominins approaching this emotional modernity would, like behaviourally modern humans today, actively seek to become the object of someone else's attention, and feel at least a glimmer of pride when approved of, and shame when disapproved of [66,116]. After a year or so these youngsters may have already been disposed to spontaneously offer something interesting or desirable to someone else, the way 14-month-old behaviourally modern humans do today, even proffering an item differing from their own preference [117]. Today's behaviourally modern Western children readily absorb and follow normative rules [118, pp. 224–225], expect others to do so, and care desperately about their own reputations [119]. When someone is trying to teach them something they not only feel pride at success, but want others to know ‘I did it!’ Equipped with sophisticated language these same children as early as 3 or 4 years of age employ flattery to cultivate the goodwill of others [120]. By early adulthood, behaviourally modern humans find it so pleasurable to talk about themselves that among contemporary Westerners, it stimulates the same neural regions as anticipating something delicious to eat would [121]. This concern with presentation of self, reputation and impressing others may fuel tendencies to register the intentions and preferences of others who are modelling behaviour and then conform. This may explain why human children, but not other apes, do not just imitate demonstrators, but sometimes ‘over-imitate’ them, adding all the same ritualized bells and whistles even if these exceed procedures needed to accomplish a task [118,122,123]. Acute sensitivity to the intentions, thoughts and preferences of others, eagerness for their approval, and a rush of dopamine and other neurochemical rewards when sensing approval, add new dimensions to social learning.

Primatewide, youngsters learn to conform to social rules while growing up, for example internalizing proper etiquette for approaching a dominant group member. But human children display special eagerness to ingratiate themselves with others and internalize their preferences, adding subjective dimensions to this quest. Evolutionary psychiatrist Randolph Nesse hypothesizes that runaway social selection favouring self-consciousness and concern with reputation in creatures already interested in mentalizing what others think explains why our ancestors evolved the internal self-monitoring known as ‘a conscience’ [124,125].

Whether or not such ingratiating tendencies encourage humans to behave in fair, generous, or civic-minded rather than more self-serving ways probably depends more on socioecological contexts and immediate goals than on what are sometimes taken to be hard-wired moral sensibilities [113,126,127]. As early as six months, long before language, infants exhibit preferences for helpful versus hurtful others [127]. However, it is unclear how prosocial versus self-interested such preferences are. In experiments simulating *voir dire* in an imaginary courtroom, Melnikoff & Bailey [128] asked adult subjects who they would prefer in the jury, depending on whether they served as lawyer for the defence or for the prosecution. The researchers were struck by how conditional on peoples' current goals their preferences for moral versus immoral actors could be. Whatever standards prevail, quests to demonstrate mental and behavioural responses conforming to others’ preferences pave the way for internalizing group norms [129] and for behaviour that others consider ‘moral’ [89,130]. It is exactly this third party perspective that is strikingly lacking in non-human primates [131], who otherwise exhibit various building blocks of morality [132,133]. Other primates may conform to local traditions, but they do not seem to care if *others* do so or not, and even unusually prosocial primates such as marmosets do not manage their reputations by increasing helping efforts when observed by others [131]. Humans, being able to represent how their behaviour appears to others (perhaps as a result of their great ape cognitive heritage) [86], appear distinctively motivated to care about their reputations. The same would be advantageous for marmosets too, but they may simply not be able to cognitively represent how they appear to others.

## 4. Conclusion

Across taxa, longer spans of post-weaning or post-fledging dependence are predictable corollaries of cooperative breeding. In the case of the cooperatively breeding apes in the line leading to the genus *Homo*, reliance on care and provisioning from alloparents as well as parents conditioned dependent immatures to develop un-ape-like eagerness to monitor and care about the intentions of others, mentalize what they were thinking and feeling, and seek to ingratiate themselves with them, leading to the expression and refinement of otherwise latent ape potentials. This novel context for development and social learning coincided with directional social selection [35,134] favouring youngsters best at ingratiating themselves with protectors, mentors and providers. By 2 Ma this combined process of development-plus-social selection was already contributing towards the emergence of ape cognitive and emotional phenotypes very different from those among our Last Common Ancestors with chimpanzees and other apes.

Without any foresight on Mother Nature's part concerning how important questing for intersubjective engagement and escalating concerns with reputations would eventually turn out to be, *H. erectus* infants would have been conditioned to monitor and care about what others were thinking, including thinking about them, and rewarded for internalizing their preferences in ways others apes were not. Reputational concerns make having a conscience increasingly useful. Long before the emergence of *anatomically modern* big-brained humans by 300 000 years ago [135], or before behaviourally modern humans with symbolic thought and language, these emotionally different apes were already eager to appeal to and help others. Furthermore, observation of humans today suggests that these tendencies emerge early and in both sexes, with girls if anything better able to interpret others' expressions and feelings than boys [136].

By the Late Pleistocene, when cooperative hunting of big game, division of labour and sharing of food became important, hominins of both sexes must *already* have become predisposed to read the intentions of others in order to coordinate with and perhaps help them [1,2,137,138]. By the time coordinated hunting of large animals was established in the human repertoire—whether by 400 000 years ago as in Tomasello's reconstruction or closer to Chris Boehm's ‘magic number’ [89, p. 313] of 250 000 years ago—it was probably accompanied by ‘punitive social selection’ against stingy or overly domineering men, as documented for most well-studied hunter–gatherer societies [89, p. 164; 90,130]. If so, these members of the genus *Homo* would have already become motivationally very different from their more self-centred, solipsistic ape ancestors. In Boehm's account, sanctions against bullies could extend to exile or even execution, pressuring group members to conform and adopt normative ‘moral’ behaviour. But with internalization of norms already underway, archaic humans were, from an early age already sensitive to what others felt and thought about them, concerned about personal reputations, and eager to cooperate. They were preadapted to internalize ways of behaving and expressing themselves that others preferred.

With higher quality food sources and with multiple provisioners continuing to buffer weanlings from recurring shortages, the grey ceiling limiting energy available for brains was raised. The stage was set for these *emotionally modern* early humans to meet Late Pleistocene social and ecological challenges in ways that would favour the evolution of even more costly, anatomically modern, brains. Accompanying motivations would also lead to the emergence of more sophisticated modes of vocal communication that would vastly expand both the ability to learn from multiple others (via gossip and teaching) and the reach and importance both of normative ways of doing things [18] and of reputations. Such processes would stress conformity and further favour the internalization of group norms and human indoctrinability, hallmarks of *behaviourally modern* humans.

## Data accessibility

This article has no additional data.
